# American ginseng modulates pancreatic beta cell activities

**DOI:** 10.1186/1749-8546-2-11

**Published:** 2007-10-25

**Authors:** Zonggui Wu, John Zeqi Luo, Luguang Luo

**Affiliations:** 1 Department of Cardiology, Second Affiliated Hospital, Second Military Medical University, Shanghai, 200003, China; 2 PLME Department of Medicine, Brown University, Providence, RI 02912, USA; 3 Center for Stem Cell Biology, Department of Research, Roger Williams Hospital, Providence, RI 02908, USA

## Abstract

The mechanism of the beneficial effects of *Panax quinquefolius *(*Xiyangshen*, American ginseng) on diabetes is yet to be elucidated. Recent studies show that *Panax quinquefolius *increases insulin production and reduces the death of pancreatic beta cells. Mechanism studies indicate that *Panax quinquefolius *improves cell's immuno-reactivity and mitochondrial function through various factors. Clinical studies show that *Panax quinquefolius *improves postprandial glycemia in type 2 diabetic patients. Further studies to identify the component(s) of *Panax quinquefolius *linked with pancreatic islets/beta cells *in vitro *and *in vivo *are warranted for better understanding of the full effects of *Panax quinquefolius*.

## Background

Increasing steadily in case numbers, diabetes mellitus has become a devastating illness with significant morbidity and mortality around the globe [1,2]. There are three types of diabetes, namely type 1, type 2 and gestational, with type 2 being the most common one. Type 1 juvenile diabetes is caused by auto-immune disorders leading to extensive destruction of the insulin-producing beta cells in the islets of Langerhans in the pancreas [3]. While type 2 adult-onset diabetes is related to insulin resistance, its cause remains poorly understood. All type 1 and some of type 2 diabetic patients require daily insulin shots. Either replacement of destroyed beta cells via islets or pancreas transplant is sometimes necessary [4].

In China, ginseng root has been used for millennia as a tonic to increase vitality [5,6]. With the recent significant increase in the usage of herbal remedies in the United States [7], attention has been focused on the two most popular types of ginseng, namely *Panax quinquefolius L*. (*Xiyangshen*, American ginseng) and *Panax ginseng CA Meyer *(*Renshen*, Asian ginseng) [3,8,9]. According to Chinese medicine theory, the two ginseng types possess different properties and display different effects. *Panax ginseng *increases blood flow and decreases fatigue. Experimental studies indicate that *Panax ginseng *alleviates oxidative stress generated by diabetes through the inhibition of lipid peroxidation [10]. On the other hand, *Panax quinquefolius *has anti-aging effects, aids digestion [8] and has anti-hyperglycemic effects. Clinical research studies demonstrate that *Panax quinquefolius *may improve psychological conditions, immune function and conditions associated with diabetes (Table 1). Overall, *Panax quinquefolius *appears to be well tolerated, although caution is advised about concomitant use with some pharmaceuticals such as warfarin, oral hypoglycemic agents, insulin and phenelzine [11]. Historical treatment of diabetes with Eastern medicine includes integration of ginseng root extracts in tonic form into the diet. While *Panax quinquefolius *has been shown to facilitate anti-hyperglycemia, it is important to investigate whether it improves pancreatic beta cell activities and whether it intervenes in various factors that may cause beta cell dysfunction and death. This review will describe and discuss a possible mechanism of anti-hyperglycemic effects of *Panax quinquefolius *by modulating pancreatic beta cell activities.

**Table 1:** Effects of American ginseng vs. those of Asian ginseng

*Panax quinquefolius L*. (*Xiyangshen*, American ginseng)	*Panax ginseng CA Meyer *(*Renshen*, Asian ginseng)
Anti-aging	Increases blood flow
Anti-hyperglycemic	Decreases fatigue
Aids digestion	Stimulates the nervous system
Reduces stress	Anti-inflammatory agent
Enhances memory	Aids insomnia
Improves sexual function	Anti-depression

## Ginseng components

The main active components in ginseng responsible for ginseng's medical value have been identified as glycosides. Glycoside is a naturally occurring substance consisting of a sugar and non-sugar moiety. Some glycosides belong to a family of compounds named saponins which produce froth under agitation by reducing water surface tension. A group of saponins in ginseng have been named ginsenosides and classified into subclasses as Ro, Ra, Rb, Rc, Rd, Re, Rf, Rg and Rh. These ginsenosides can be differentiated in thin layer chromatography (TLC) based on their retention factor value, which is the distance that ginsenosides travel up the TLC column [12]. *Panax ginseng *can be distinguished from *Panax quinquefolius *by using ginsenoside profiles because *Panax ginseng *contains ginsenoside Rf which is absent in *Panax quinquefolius *[13]. The anti-hyperglycemic effects of the total ginsenosides extracted from *Panax ginseng *were studied in diabetic C57BL/6J *ob/ob *mice. The results indicate that the total ginsenoside extract has significant anti-hyperglycemic and anti-obesity properties [3]. In the same animal model, it was revealed that the anti-hyperglycemic effect of ginsenoside Re was due to improved muscle metabolism and that ginsenoside improved diabetes by alleviating inflammation through increasing C-reactive protein levels [14]. Rh2, another ginseng component, lowered plasma glucose level in Streptozotocin (STZ)**-**diabetic rats in a dosage dependent manner [15]. Recent studies support the notion that ginsenosides target steroid hormone receptors, which suggests that these ginseng components act as natural steroids, offering a diverse range of pharmacological activities [16].

## *Panax quinquefolius *facilitates normalization of hyperglycemia

*Panax quinquefolius *lowers blood glucose in diabetic patients [9], increases pancreatic beta cell insulin production and secretion, and prevents beta cell apoptosis [17]. The results also show that the effects of *Panax quinquefolius *in stimulating insulin production/secretion and anti-apoptosis are dosage dependent, suggesting a biological specificity of ginseng water extracts on beta cells. A high dosage of *Panax quinquefolius *is probably required to reverse IL-1 beta induced apoptosis. According to a clinical study by Vuksan *et al*., *Panax quinquefolius *attenuated postprandial glycemia in both type 2 diabetic and non-diabetic patients. While no difference was found in postprandial glycemia between placebo and ginseng groups in non-diabetic subjects when administered together with glucose challenge, in subjects with type 2 diabetes significant reductions were observed when ginseng was taken 40 minutes before the glucose challenge [8].

## Mechanism of *Panax quinquefolius *for normalization of hyperglycemia

**Figure 1:**
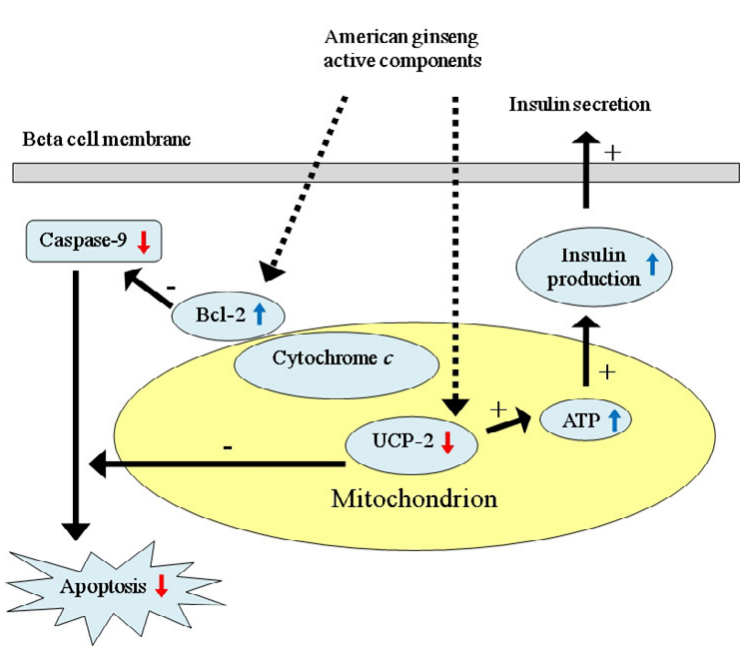
The active components of *Panax quinquefolius *activate the cytochrome-*c *related Bcl-2 and inhibit UCP-2. The activation of cytochrome-*c *related Bcl-2 has negative effects on caspase-9 triggered apoptotic cascade and then apoptosis. As UCP-2 induces apoptotic cascade and decreases ATP production, the inhibition of UCP-2 has negative effects on apoptosis and positive effects on ATP production, thereby increasing insulin production and release.  : up-regulated.  : down-regulated. + : positive effects. - : negative effects.  : known actions.  : recently discovered actions.

The mechanism for the beneficial effects of *Panax quinquefolius *has not been fully elucidated. According to our findings, *Panax quinquefolius *water extracts reversed an IL-1 beta induced increase of uncoupling protein-2 (UCP-2, a mitochondrial protein which uncouples electron transport from ATP production), decreased pro-apoptotic protein caspase-9 and increased the level of anti-apoptotic protein Bcl-2. This indicates that the anti-apoptotic effects of *Panax quinquefolius *may be achieved through regulating mitochondrial UCP-2 and ATP levels, thereby influencing insulin synthesis and apoptotic cascades in beta cells [17] (Figure 1). While cell metabolism seems to be a critical factor targeted by *Panax quinquefolius*, there are other reports on additional factors targeted by ginseng such as immuno-reactivity [18]. Some other reports indicate that Rh2 lowers plasma glucose level induced by an increase of beta-endorphin secretion in STZ-diabetic rats, which activates opioid mu-receptors. The activation of opioid mu-receptors increases the expression of GLUT 4, which raises muscle and adipose tissue sensitivity to glucose stimulation, thereby promoting tissue absorption of glucose, which leads to a reduction in blood glucose level [15]. Another study on STZ-diabetic rats demonstrated that ginsenoside Re relieved oxidative stress in the kidney and eyes, and reduced blood glucose, cholesterol and triglyceride levels. These findings suggest that different components of ginseng may act together to achieve its overall therapeutic effects [19]. Protopanaxatriol (PPT), one of the ginsenoside metabolites, increases GLUT4 and improves insulin resistance [20]. Wang *et al*. reported that *Panax quinquefolius *exerted an anti-lipolytic effect on type 2 diabetic conditions through a signaling pathway different from that of insulin, suggesting that *Panax quinquefolius *not only affects pancreatic cells through increasing insulin production and cell viability, but also through creating an anti-lipolytic effect and targeting glucose receptors to counter hyperglycemia [21]. Not only has ginseng root been found to have positive effects, but also ginseng leaf and berry extracts have been found to have potent anti-oxidant activities against free radicals that are excessively produced in diabetic complications [22]. Indeed, these findings are merely the tip of the iceberg in terms of the information required for elucidating the mechanism for *Panax quinquefolius *to improve hyperglycemia and treat diabetes.

### Panax quinquefolius improves beta cell insulin production

The inability to produce adequate amounts of insulin is a factor attributed to beta cell dysfunction. Possible causes include a disorder in beta cells preventing the production of insulin and the death of the insulin producing beta cells. When water extract of *Panax quinquefolius *was administered to IL-1 beta cultured dysfunctional pancreatic beta cells, insulin production was significantly increased in dysfunctional beta cells [17]. Apart from many other factors, lack of insulin production is primarily due to deficiency of ATP generated by mitochondria [12,23]. *Panax quinquefolius *appears to enhance ATP production. An inverse correlation between the amount of ATP and the level of UCP-2 reveals an effect of *Panax quinquefolius *on beta cell function. UCP-2 down-regulates insulin secretion by preventing ATP production [23-27]. UCP-2 belongs to a family of anion mitochondrial transporters linked to negatively regulated metabolism. UCP-2 has been found to be involved in anti-hyperinsulinemia after the detection of the gene in pancreatic beta cells [27]. Cells induced with IL-1 beta exhibit a much higher level of UCP-2, indicating that a high level of UCP-2 is harmful to the cell. Cells with a high level of UCP-2 tend to have lower survival rates [28]. *Panax quinquefolius *significantly reduces the level of UCP-2 and increases ATP in the mitochondrion. *Panax quinquefolius*'s ability to regulate UCP-2 may explain how it increases beta cell function and viability.

### Panax quinquefolius protects beta cells from apoptosis

A gradual loss of beta cells due to apoptosis (i.e. programmed cell death) significantly hinders insulin production and inhibits cell viability. During apoptosis, cells shrink; chromatin condenses; DNA is cleaved into pieces at internucleosomal regions [18]. The cell breaks down into small pieces which are then absorbed by neighboring cells without causing inflammation. A proactive way to increase beta cell viability is to decrease apoptosis level in order to retain the cell population and increase insulin production. Apoptosis is mediated by many factors, one of which is the Bcl-2 proto-oncogene [29]. Bcl-2 protects the cell against apoptosis by preventing activation of the caspase cascade. The caspase cascade involves translocation of cytochrome *c *from the mitochondrion to activate caspase-9 and then caspase-3, initiating the proteolytic cascade essential for apoptosis [30]. Previous studies suggest that *Panax quinquefolius *regulates pancreatic beta cell apoptosis through mediating the levels of Bcl-2 and capase-9 [17]. In a study of Bcl-2 and caspase-9 levels in response to *Panax quinquefolius *in damaged cells showed that *Panax quinquefolius *significantly up-regulated the levels of Bcl-2 and down-regulated levels of caspase-9 expression [17]. IL-1 beta treated cells without ginseng treatment showed a one-fold higher expression of pro-apoptotic factor caspase-9 than that of cells with ginseng treatment [17]. *Panax quinquefolius *may activate the mitochondrial membrane protein Bcl-2 to prevent cell apoptosis. By regulating the UCP-2 level, *Panax quinquefolius *increases ATP production, thereby increasing insulin production, meanwhile it prevents apoptosis by reducing caspase-9 activation and raising levels of Bcl-2 [17]. Further research is thus warranted in analyzing all aspects of *Panax quinquefolius*'s role in benefiting pancreatic beta cells.

## Uncertainties

Due to a lack of standardization in the herbal medicine industry, it is not certain whether the beneficial effects on hyperglycemia hold true for all *Panax quinquefolius *products. It is also not certain whether different ginseng species would have the same effects on hyperglycemia. Sievenpiper *et al*. discovered that *Panax ginseng *showed both null and opposing effects on acute postprandial plasma glucose and insulin, which did not coincide with the findings with *Panax quinquefolius *[31]. Reproducible efficacy using an acute postprandial clinical screening model is necessary to standardize herbal products and to link herbal composition with efficacy in treating diabetes [32]. Further data are also required to confirm that *Panax ginseng *has the same effects on humans as it does in animal models.

## Conclusion

*Panax quinquefolius *increases cell insulin production and cell viability through mediating mitochondrial proteins and apoptosis factors. These findings confirm that *Panax quinquefolius *improves pancreatic islet functions. The components of *Panax quinquefolius *that improve pancreatic beta cell function have not been identified. The precise actions of *Panax quinquefolius *components on other human cells such as muscle cells and lipocytes under diabetes therapy are yet to be investigated.

## Competing interests

The author(s) declare that they have no competing interests.

## Authors' contributions

ZW proposed to cooperate in this project and provided information for the manuscript. JL conceived this project, collected information and wrote the first draft of the manuscript. LL worked with JL to conceive this project, collected references andinformation, wrote and approved the manuscript. All authors approved the final manuscript.
